# Developing Mental or Behavioral Health Mobile Apps for Pilot Studies by Leveraging Survey Platforms: A Do-it-Yourself Process

**DOI:** 10.2196/15561

**Published:** 2020-04-20

**Authors:** Philip I Chow

**Affiliations:** 1 Center for Behavioral Health and Technology Department of Psychiatry and Neurobehavioral Sciences University of Virginia Charlottesville, VA United States

**Keywords:** app, mental health, mHealth

## Abstract

**Background:**

Behavioral health researchers are increasingly recognizing the potential of mobile phone apps to deliver empirically supported treatments. However, current options for developing apps typically require large amounts of expertise or money.

**Objective:**

This paper aims to describe a pragmatic do-it-yourself approach for researchers to create and pilot an Android mobile phone app using existing survey software (eg, Qualtrics survey platform).

**Methods:**

This study was conducted at an academic research center in the United States focused on developing and evaluating behavioral health technologies. The process outlined in this paper was derived and condensed from the steps to building an existing app intervention, iCanThrive, which was developed to enhance mental well-being in women cancer survivors.

**Results:**

This paper describes an inexpensive, practical process that uses a widely available survey software, such as Qualtrics, to create and pilot a mobile phone intervention that is presented to participants as a Web viewer app that is downloaded from the Google Play store. Health researchers who are interested in using this process to pilot apps are encouraged to inquire about the survey platforms available to them, the level of security those survey platforms provide, and the regulatory guidelines set forth by their institution.

**Conclusions:**

As app interventions continue to gain interest among researchers and consumers alike, it is important to find new ways to efficiently develop and pilot app interventions before committing a large amount of resources. Mobile phone app interventions are an important component to discovering new ways to reach and support individuals with behavioral or mental health disorders.

## Introduction

### Background

Considering the worldwide burden of mental and behavioral health disorders, researchers are recognizing the importance of exploring alternative treatment delivery options, given the following drawbacks of in-person treatment: financial cost [1], time investment [2], social stigma [3], and a shortage of trained health care providers [4-6]. Increasingly, health researchers are discovering the importance of using mobile phone apps to fill a critical health care gap [7-9]. In contrast to in-person health delivery, in many circumstances, app interventions can increase accessibility to users [10,11], are more affordable, and can offer a more efficient use of time. As literature on the efficacy of behavioral and mental health apps continues to flourish [10,12,13], developing and piloting apps remains a significant challenge for many health researchers who lack any requisite experience in native app coding and app development. Having a method to quickly develop, iterate, and pilot app interventions can lead to a more agile mobile health (mHealth) research lifecycle [14].

### Options for App Development

Currently, 2 options generally available to health researchers who wish to develop and test their own app interventions are to (1) write their own software code or (2) have someone else (usually a software engineer) generate the software code. As many health researchers lack expertise in software coding, an attractive option is to procure the services of someone who does. Some academic settings make it possible to leverage the programming services of students (eg, engineering students), given that the number of individuals with programming experience has increased in recent years. Despite these potential options, many health researchers still opt to pay private app developers that primarily focus on building native apps that run on either the Android or iPhone operating systems. Thus, many health researchers who are skilled in intervention development and validation, and are interested in piloting their own intervention apps, never get the opportunity to test their ideas because of financial constraints. This limits the field of app development to researchers with funding, private for-profit corporations, and software engineers. Therefore, it is not surprising that despite a proliferation of health apps available to the public, only a small fraction have been empirically tested and validated [13,15].

In addition to cost, paying a third-party developer to build a native app has additional drawbacks. First, native apps must be adapted to the context of each operating system [16]. Despite some considerable strengths of native apps, such as the ability to leverage mobile phone sensors for passive data collection, they can be vulnerable to crashing because of software updates, and some of them may be incompatible with certain types of mobile phones [17]. Native software code that is developed to run on Android mobile phones cannot be reused for developing an iPhone app, and vice versa [17]. If the unique strengths of a native app are not required for a pilot research trial, researchers may want to cheaply develop and pilot an app intervention without having to commit a large amount of resources. Paying someone else to develop an app can also lead to delayed progress caused by (1) breakdowns in communication between researchers and app developers and (2) scheduling issues on behalf of developers (eg, a developer may decide to deprioritize an app if another more lucrative contract is competing for their time and attention). Additional funds are often necessary for app maintenance, data storage, and making changes to the app once the trial has begun. Thus, a significant amount of money and time can be wasted if a pilot study evaluating an app yields null findings.

### A Do-It-Yourself Approach to App Development

This paper is intended for mental and behavioral health researchers who possess content knowledge in a scientific domain (eg, depression, anxiety, and nutrition) but lack critical funding and skills in app development and coding to pilot their own app ideas. Researchers who have the ability to pay an app developer are strongly encouraged to consider that option. The purpose of this paper was to present an alternative, do-it-yourself process for developing and piloting an app by leveraging a widely available survey software, typically referred to as a Web-based app or a hybrid app [16]. In this type of app, the intervention content is built in, and hosted by, the survey platform. All data are also stored by the survey platform and are accessible only to the researcher. Whenever a user launches the app from their mobile phone, the intervention is displayed on their screen through their Web browser. The apps that can be constructed using this process are generally limited to those that administer intervention content through text, interactive exercises, audio files, and video. These apps are not able to passively collect fine-grained data through mobile phone sensors or execute complex sensor-driven interventions (eg, a just-in-time adaptive intervention [18]). Researchers who are interested in having these functionalities in their intervention should consider a native mobile phone app. Although there are several industry platforms for prototyping apps (eg, InVision and Proto.io), many of these are tools for designing an app rather than building an app for data collection in a pilot study. One key advantage of using a secure Web platform such as Qualtrics for highly sensitive data (Qualtrics, Provo, Utah) and Research Electronic Data Capture (REDCap; Vanderbilt University, Nashville, Tennesse) is that, in addition to providing researchers with full control over the intervention development process, these platforms are often compliant with local laws and regulations (eg, Health Insurance Portability and Accountability Act [HIPAA]) for storing protected health information data.

The app intervention discussed in this paper, iCanThrive, is meant to serve as an example of the app building steps provided. It was built using the Qualtrics survey platform for highly sensitive data portal and teaches evidence-based skills grounded in cognitive therapy, acceptance-based therapies, and positive psychology. The app contains brief, intuitive exercises that focus on identifying and challenging distorted thoughts, problem solving, reducing worry, reducing stress through breathing and mindfulness exercises, fostering gratitude, promoting values, savoring positive experiences, and increasing emotional awareness. It was designed to be deployed on users’ mobile phones when and where it is needed. The app is available for download on the Google Play store for US Android users. Google Play is the official app store for the Android operating system and is available in over 145 countries. In a recently completed pilot, the app was downloaded and used by 23 cancer survivors. The participants did not report any issues in downloading or using the app. A paper describing the acceptability and preliminary efficacy of iCanThrive is currently under revision [19].

## Methods

Five phases were identified to developing a Web-based app (Table 1), which is browser based and hosted in Qualtrics. The phases outlined below are not exhaustive, and researchers are encouraged to consult with the appropriate individuals at their institution (eg, data security experts and institutional review board members) before determining how best (and whether) to undertake this work at their institution. The general phases of the app development process are presented first, followed by a description of how these phases were reflected in the development of the iCanThrive app.

**Table 1:** General phases of app development using Web-based survey software.

Phase	Description
Establish a theoretical framework	Using existing models of electronic/mobile health, mental health, and cancer to develop a theory-informed intervention.
Wireframe and develop content	Begin to develop content and create a visual representation of how content will be delivered through the app.
Build the intervention in a survey platform	Insert content in an existing survey development platform (Qualtrics), using existing tools to create an interactive and app-like user experience. Test frequently.
Post app on the Google Play store	Using the survey Web link, post the app on the Google Play store for download.
Data quality and output	Before beginning a pilot study with actual participants, ensure that the data are being collected and relevant information (eg, log-ins and session durations) is being collected.

## Results

### Phase 1: Establish a Theoretical Framework

The first step is to ground the intervention in a theoretical framework based on models of behavioral change [20-22]. It is recommended that researchers incorporate existing electronic health/mHealth intervention models into their conceptualizations. Existing models of internet interventions [23] and mobile interventions, such as the behavioral intervention technologies model [24], can be useful in developing the theoretical framework for app-based interventions. For example, researchers who are designing an intervention that is informed by the theory of planned behavior [20] may seek to modify people’s beliefs about the degree to which they have control over some aspect of their behavior (eg, exercising and smoking). Combined with an mHealth intervention model [24] that promotes the use of brief and targeted app sessions, they may decide to provide brief educational content that can be digested by users in short periods or add interactive components (eg, videos) to enhance user experience.

### Phase 2: Wireframe and Content Development

**Figure 1:**
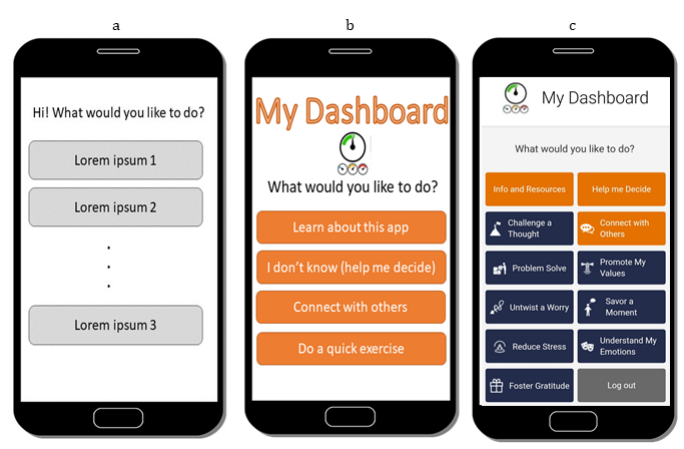
Examples of 2 previous wireframes for the iCanThrive main dashboard page using PowerPoint. From left to right: (a) early iteration sketch, (b) a more visually appealing iteration, and (c) actual screenshot of the iCanThrive dashboard page.

A wireframe is a visual guide of the skeletal framework of the app. As highlighted by others [25], the purpose of creating a wireframe is to determine how best to deliver and meter content across different app pages to achieve the desired effect. In this phase, researchers develop content and determine how best to present the content in a way that is engaging, intuitive, and achieves the purpose of the intervention [23,24]. Researchers can create a wireframe from widely available software, such as PowerPoint (Microsoft Office, Redmond, Washington) and InVision (InVisionApp Inc, New York, New York), or they may choose to draw their wireframe using a paper-and-pencil method. There is also a range of affordable and user-friendly wireframing tools that researchers can search for on the Web, such as Adobe Xd (Adobe, San Jose, California) and Lucidchart (Lucid Software Inc, South Jordan, Utah). Although researchers should expect to iterate during the app construction phase, the wireframe provides an essential blueprint from which to start. Researchers may wish to support users with an overview of the app, how to use it, and expectations users should have of the app. For example, by providing a help option from a main page, researchers can ensure that users can easily access instructions by promoting an intuitive user interface [26]. Another way of promoting user interface is by embedding email links within the app itself to support users and to be responsive to their questions and concerns. Examples of a wireframe process can be seen in Figure 1. The sketching and outlining of app pages allow for quick iterations of existing prototypes until a desired form is achieved.

**Figure 2:**
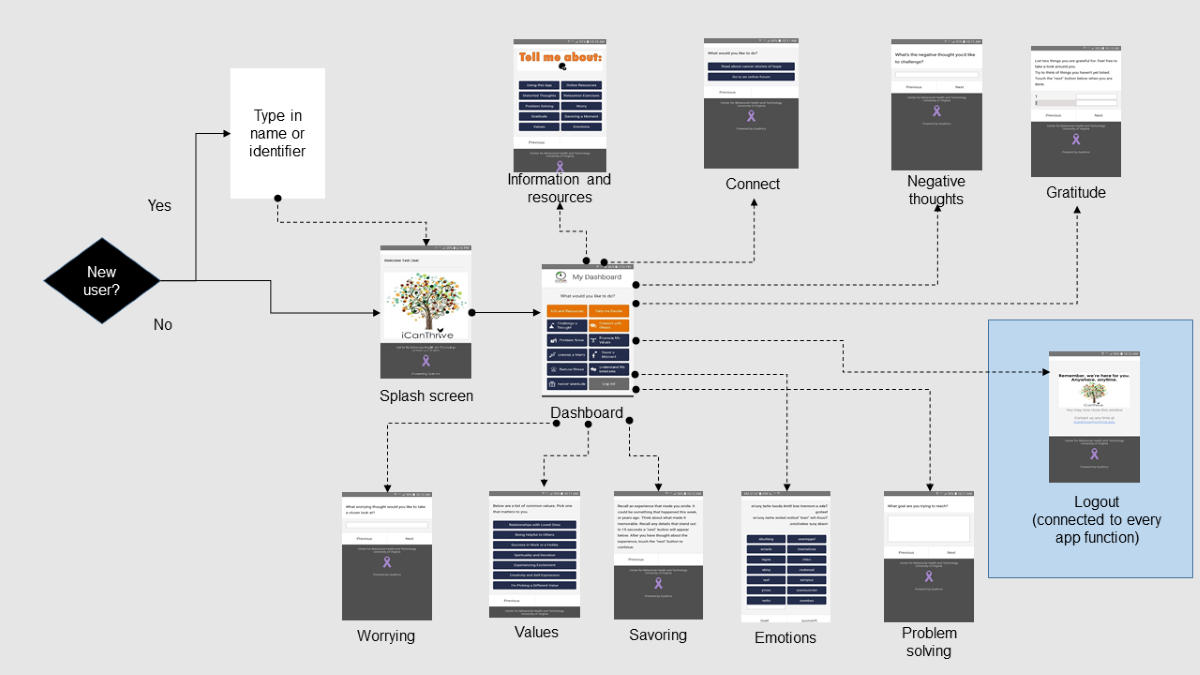
Example of a schematic that tracks how a user can navigate the app by showing how the front app pages and functions are connected.

In addition to prototyping the content and layout of app pages, researchers are encouraged to track how different pages are connected within the app (Figure 2). This enables a high-level perspective of how the app functions and the paths a user can adopt as they navigate the app.

### Phase 3: Build the Intervention in a Web Survey Platform

The next phase involves actually building the intervention in a secure Web survey platform. The iCanThrive intervention was built in the Qualtrics survey platform designed to collect and store highly sensitive data. Universities often have subscriptions to survey platforms such as Qualtrics and REDCap, which have similar functions and are typically free for their faculty and students to use for research purposes. Researchers should inquire about the options at their institution and become familiar with using the survey platform available to them. If the intention is to collect sensitive data, researchers should use a survey platform that is secure and compliant with security, privacy, and ethical standards (eg, HIPAA).

**Figure 3:**
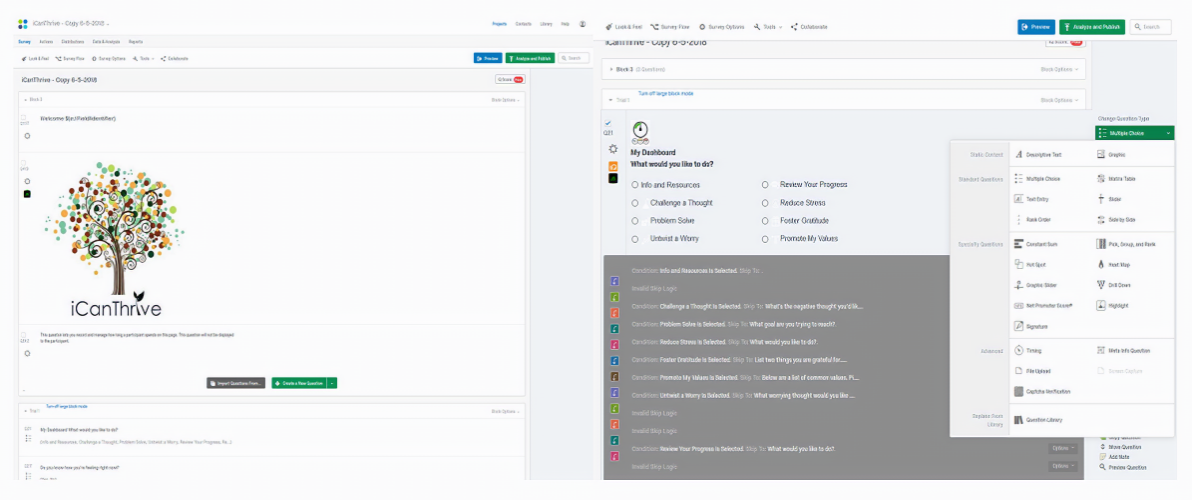
Screenshots of the iCanThrive splash screen and dashboard pages.

Similar to other survey platforms, Qualtrics has preset options for how to display content through different question types. As it is designed to create surveys rather than interventions, Qualtrics naturally labels a new item as a *question*, which is then grouped by *blocks* (Figure 3). Thus, each app page in an intervention (eg, iCanThrive) is actually a different Qualtrics *question* embedded in a single block (Figure 3). An intervention is essentially an elaborate survey with embedded skip and display logic and other features available in the survey platform. Every time a user launches the app, they are completing a Qualtrics survey, which is then stored by the survey platform. Researchers are encouraged to explore the different preset options for creating and displaying content (see Figure 3 for a list of available options in Qualtrics). For example, the *Multiple-Choice* option is useful for creating a checklist of options to which users can respond (Figure 3), whereas the *Graphic* option is convenient for inserting educational figures or logos. This option can be used to create a splash screen, which contributes to an authentic app feel that a user sees when they launch the app from their phone. Question types should be chosen strategically to fulfill the purpose of each app page. For example, the *Text Entry* option is useful for allowing users to freely respond or generate their own examples using their phone’s keyboard. A text entry field may also be used to have a user type in a unique subject identifier every time they launch the app, which enables researchers to track the number of app launches for each user. Tracking usage in this way can also allow researchers to monitor app launches across different devices (eg, if a user gets a new phone). After choosing a preset option and inserting content, the *Preview Question* option is helpful for evaluating the page to achieve the desired format.

In addition to creating content, Qualtrics has useful tools for connecting different pages within the intervention. For example, after creating a new page, researchers are able to link that page to other existing pages through the *Add* *Skip Logic* option. This allows researchers to tailor intervention content based on a user’s responses. For example, researchers can directly link an app landing page to other pages within the app. In addition, researchers are able to create customized navigation routes through the *Add Display Logic* option found in each Qualtrics question. Researchers can use this function to present users with different pages based on their responses, such as providing additional educational content if a user indicates that they are struggling with some aspect of the intervention. Once again, researchers are encouraged to explore different options to create the type of navigation they desire within the intervention.

**Figure 4:**
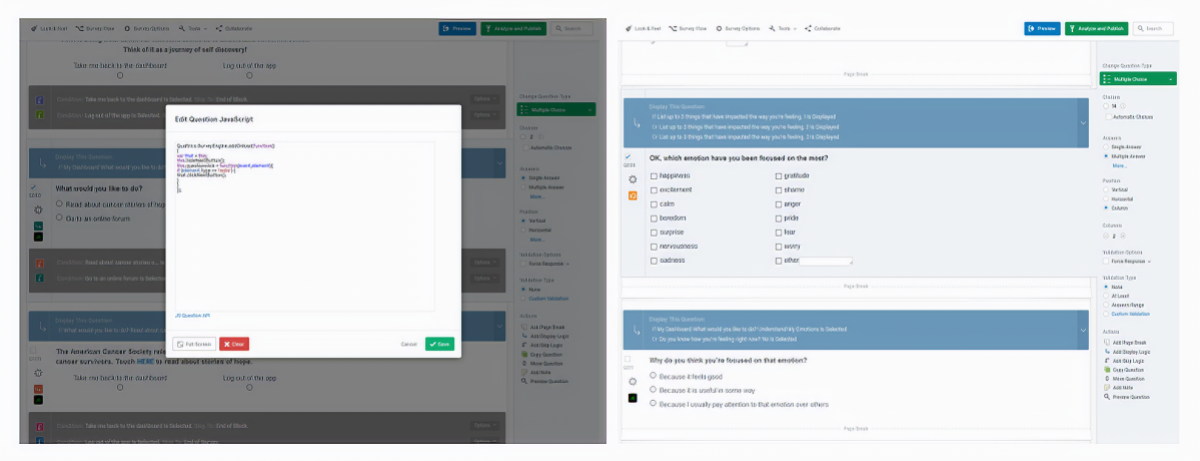
Screenshots of the custom JavaScript and using the multiple-choice question type to create intervention content in the iCanThrive app.

Qualtrics has additional features that enable researchers to enhance a user’s experience through programmable code (eg, JavaScript). For example, in the preset version of Qualtrics, after indicating a response, individuals are asked to select a *next* button to proceed to the next page. Researchers who have some rudimentary background in JavaScript can directly insert their own code in a Qualtrics question that will enable users to bypass this step, allowing them to automatically jump to the appropriate page when they make a selection to a multiple-choice question (see Figure 4 for the JavaScript code that was used). It should be noted that the use of programmable code is optional in the app process outlined in this paper. Health researchers who do not have the time to acquire any coding skills are encouraged to bypass the use of code. Qualtrics also enables researchers to change the overall presentation of the intervention through the *Look & Feel* tab, where researchers can enhance user experience by inserting customized style sheets (eg, to change the colors of response buttons) or use existing drop-down options such as selecting different color schemes. It is important to note that researchers who lack the requisite experience to make such changes (eg, creating customized style sheets) yet are interested in exploring these options, may wish to pay a Web developer to enact the desired changes. Researchers are strongly encouraged to test their app frequently before conducting a trial with actual cancer survivors. As the functions of the app are executed within the robust Qualtrics survey platform, health researchers may wish to evaluate the app based on aesthetic qualities (eg, color scheme, font sizes, and graphics), intuitiveness of using and navigating the app (eg, sophistication of language in the app and use of too many required text fields that may impede progress), and whether the app pages are connected in the intended ways (eg, skip logic redirects the user to the appropriate page in the app). Researchers should consider what resources are available to them in evaluating the app. For example, students can be asked to evaluate whether the app pages are linked in the appropriate ways. End users (eg, cancer survivors) may be asked to trial the app to evaluate aesthetic qualities and the language used in the app pages. Though it is outside the scope of this paper, there is abundant literature on involving cancer populations in the development and testing of interventions [27-31].

### Phase 4: Post the App on the Google Play Store

**Figure 5:**
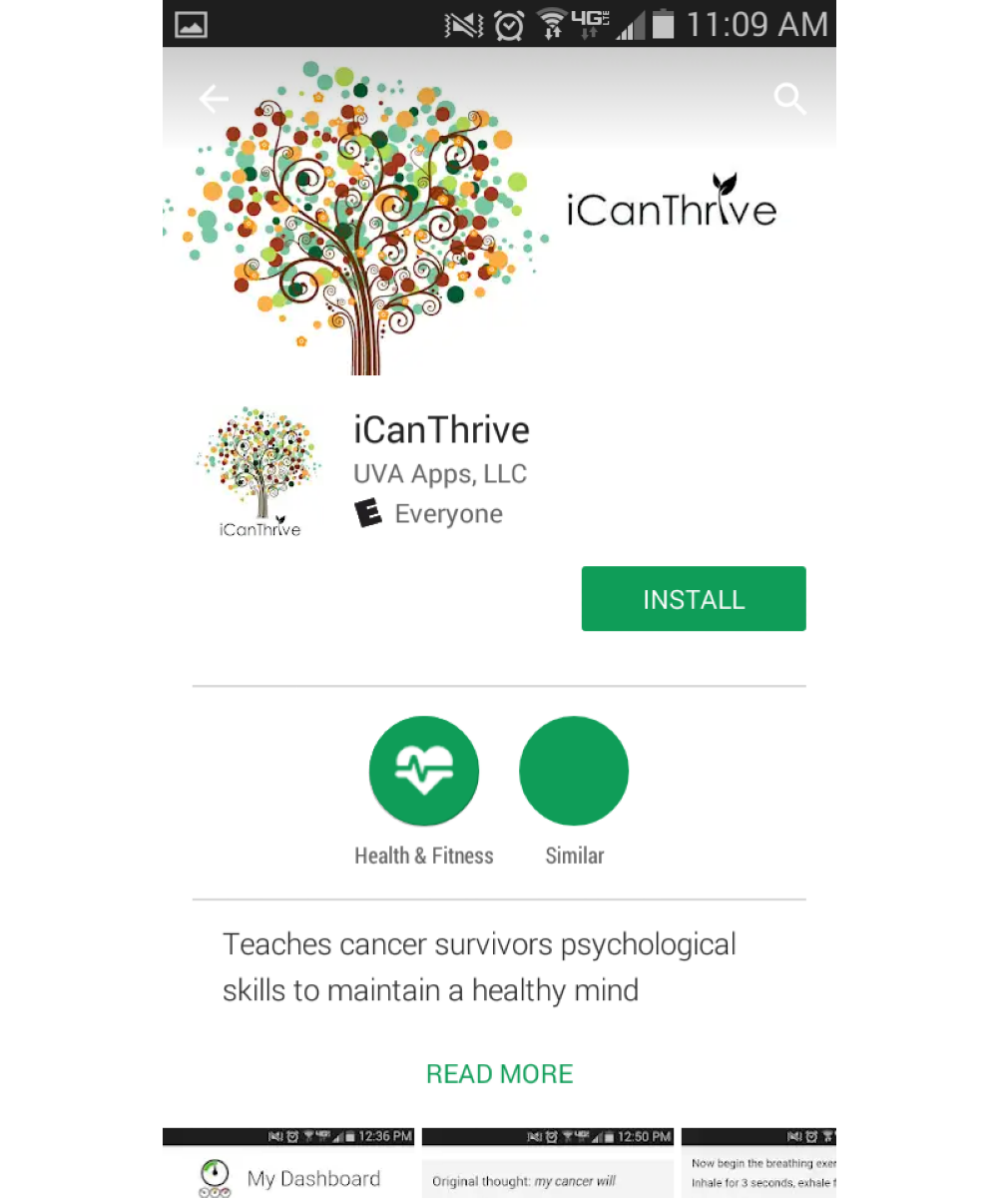
Screenshot of the iCanThrive app on the Google Play store.

Qualtrics provides a unique URL for each survey that is created in its platform. The URL is the unique Web address that is linked to that survey. The URL of the survey that contains the intervention content can be used to post a Web viewer app to the Google Play store for user download (Figure 5). As the process of posting an app on the Google Play store is beyond the scope of this paper and is the same for all types of Android apps, researchers are encouraged to learn about this process through Google’s support pages or other reputable sources. To post an app requires a one-time registration free (US $25 as of 2018), and Google Play allows for the release of apps to select groups of users as alpha or beta tests.

### Phase 5: Data Quality and Output

**Figure 6:**
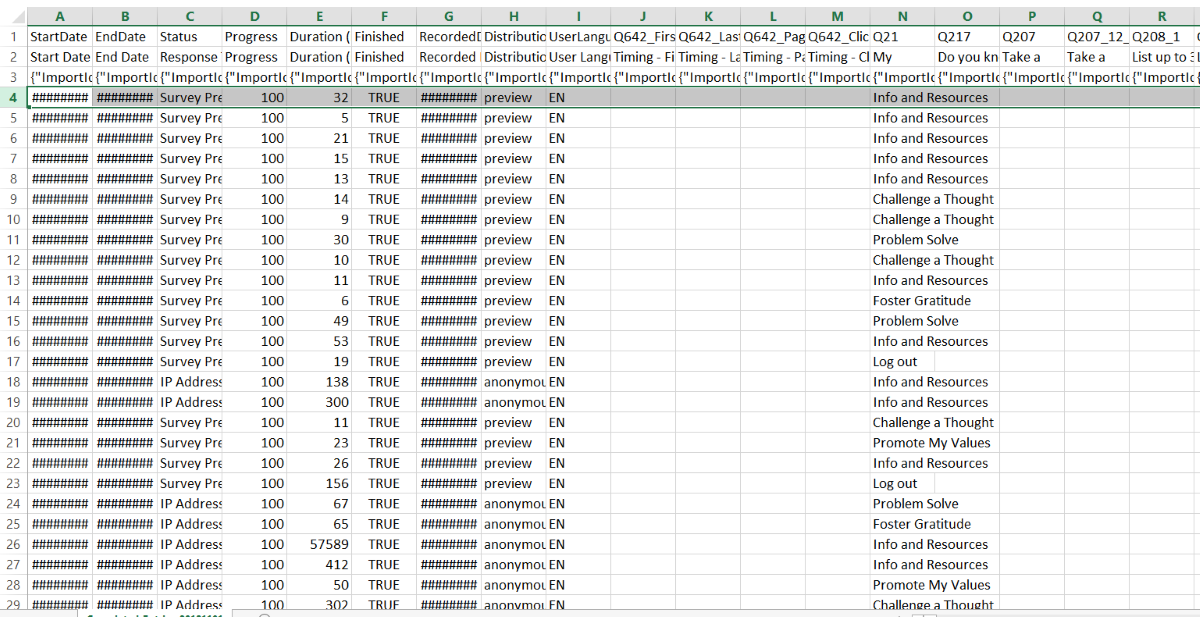
Screenshot of a hypothetical data spreadsheet.

Similar to other survey platforms, Qualtrics can export data in a convenient spreadsheet on demand (Figure 5). It should be noted that researchers should be aware that there are 2 sources within Qualtrics from which to download data: (1) recorded responses, which is composed of sessions in which a user completes a session by *submitting* their responses, and (2) responses in progress, which is composed of sessions in which a user begins a session but does not submit their response or prematurely closes their Web browser (these types of data should be downloaded by the researcher every month of the trial, as responses in progress are not saved in Qualtrics in perpetuity). As seen in Figure 6, data are presented in a wide table format, whereby columns represent different variables found within the intervention and each row represents a separate log-in from a user. Entries are presented in chronological order, although researchers may choose to represent the data in whatever way suits them best. Importantly, information such as app launches can be easily calculated from the first few columns of the data spreadsheet, as for every log-in attempt, Qualtrics records the date, time, and duration in seconds. It should be noted that because of its design as a survey platform, the order of columns in the data spreadsheet corresponds to how the question items are ordered in the actual survey. However, researchers who wish to examine how users responded to specific intervention content (within each app launch) and navigated through the app may still obtain this information by sorting through the columns. For example, column *N* in Figure 6 contains what module users selected when they first encountered the landing screen in iCanThrive, and by scanning the rows of the data file, it is possible to understand how users navigated through the app at each log-in.

### Example: iCanThrive

#### Phase 1: Establish a Theoretical Framework

The iCanThrive app was intended for users to launch where or when they desired and follows a cognitive behavioral therapy (CBT) framework, whereby patients acquire skills by practicing them in daily life. This approach was informed by findings that suggest that people use apps in short and frequent bursts [32] and was believed to be particularly relevant to cancer survivors given the considerable amount of time of cancer treatment [2] and its impact on leisure time [33]. The model of internet interventions [34] informed the use of video content and exercises to promote mental health. Thus, iCanThrive was designed to increase mental well-being by delivering brief interactive exercises that cancer survivors could perform in daily life, when and where they were needed.

#### Phase 2: Wireframe and Content Development

**Figure 7:**
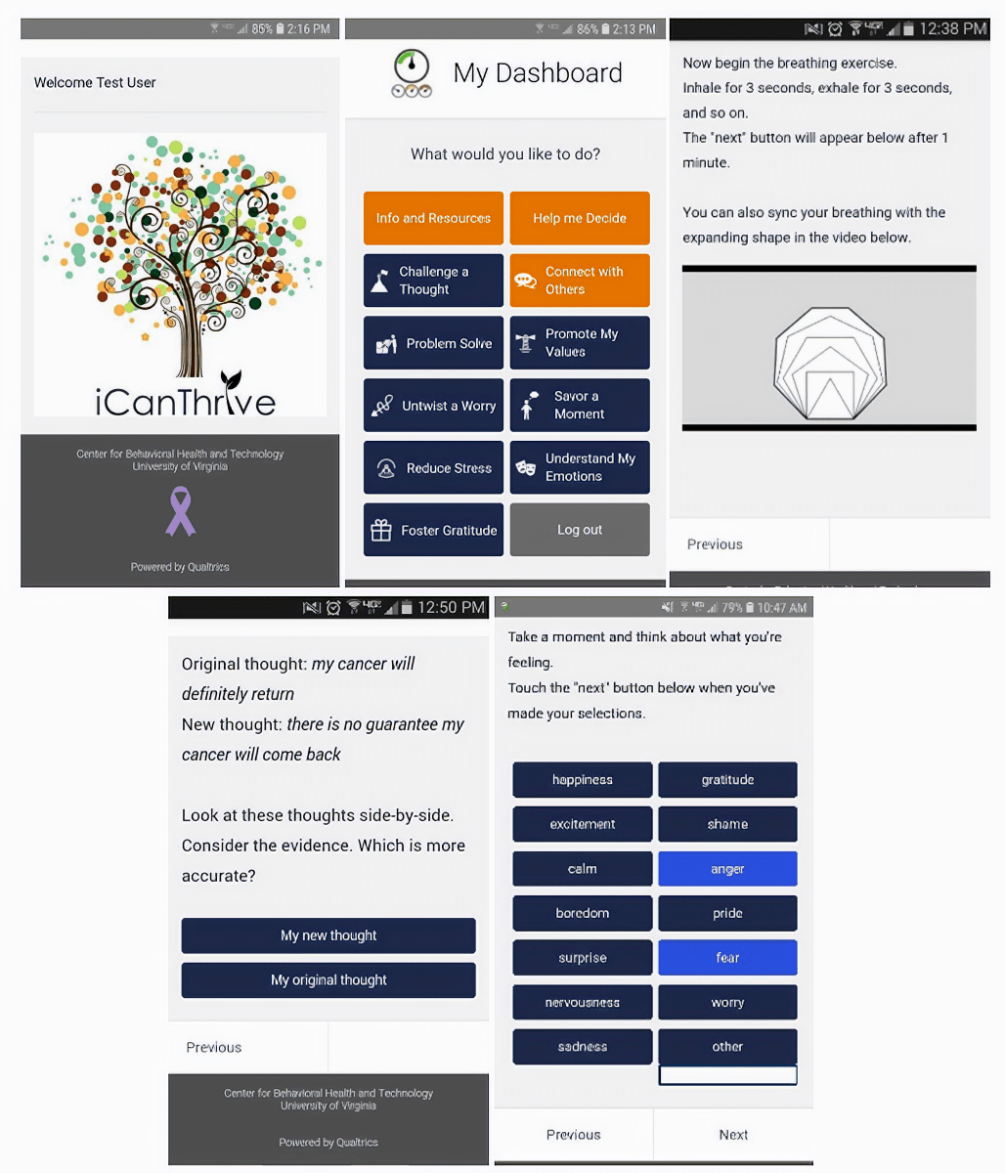
Screenshots of iCanThrive app pages.

The iCanThrive app was developed with a detailed wireframe that informed the development and placement of each app page (Figures 1 and 2). Upon launching the iCanThrive app, users are presented with a brief splash screen that contains the app logo (Figure 7, left), before automatically directing them to the *My Dashboard* screen (Figure 7, second from left). From this screen, users can navigate freely by selecting the appropriate option on their phone screen. They can access any of the 8 intervention modules, learn more about the psychological constructs the app targets, or be guided to 1 of the 8 intervention modules based on a series of questions that assess the user’s current state.

The *My Dashboard* screen was a critical component of the skeletal framework of the app, a central point from which users can easily navigate. For example, if a user picks the *Challenge a Thought* module from the *My Dashboard* screen, they are guided through an interactive exercise that leads them through the steps of recording a distorted thought, classifying the thought, and ultimately generating a new alternative thought. At the end of the exercise, users are given an option to log out of the app (which submits their responses) or navigate back to the *My Dashboard* screen. Each app page in the *Challenge a Thought* module was articulated in the iCanThrive wireframe before the app was constructed and follows evidence-based approaches in CBT. An additional consideration that informed the design of the app was wanting users to complete exercises in as little time as possible. Users can complete most exercises in iCanThrive in less than 2 min. The exercises require few instructions to complete, and any instructions are clearly presented on the screen.

If a user chooses the *Info and Resources* option from the *My Dashboard* page, they are brought to a page that contains an overview of the app, how to use it, and expectations that users should have of the app. Users may also directly contact the iCanThrive research staff through embedded email links within the app itself.

#### Phase 3: Build the Intervention in a Web Survey Platform

Each app page in iCanThrive is actually a different Qualtrics *question* embedded in a single block (Figure 4). This option was chosen to create the iCanThrive splash screen, which users see every time they launch the app. The *My Dashboard* landing page in iCanThrive is linked to many other pages within the app. Users are automatically sent to the first page of the module they select (eg, *Challenge a Thought* and *Understand My Emotions*) from the *My Dashboard* page. The app also contains customized navigation routes. For example, in the *Reduce Stress* module in iCanThrive, after completing a breathing exercise, users are asked if they are more or less relaxed than they were before the exercise. If a user indicates they are more relaxed, they are sent to a page that congratulates them for their success. If a user indicates they are less relaxed than before, they are sent to a page that normalizes their experience and encourages them to try again. The use of an app to support the delivery of content and simple exercises has been shown to be effective in reducing mental health issues [35-37].

All components of the app, iterative testing, and app modifications were performed by the lead author (who is not a software developer), and therefore, there were no costs in the development of iCanThrive other than investigator time. Testing of an app page was performed after each modification to ensure there were no errors. Every page in iCanThrive went through 2 to 15 iterations, depending on the complexity of the content and interactive content. After all app pages were tested and assembled, a Web developer was [37] paid less than US $400 to make cosmetic improvements to the iCanThrive program. Researchers may wish to apply a more user-centered design to developing their own app-based interventions [38].

#### Phase 4: Post App on the Google Play Store

The iCanThrive app was posted on the Google Play store in May 2018. To further customize the app to the individual user, a local Web developer enabled a few lines of code that prompts users to write their name upon launching the app for the first time. Subsequent app launches will display the user’s name at each app launch (Figure 7), which will also be listed in the final data spreadsheet.

#### Phase 5: Data Output

As seen in Figure 6, iCanThrive data are presented in a wide table format. As each row represents an instance in which the program was initiated, a health researcher can track the number of app launches.

## Discussion

### Principal Findings

This paper is intended to provide mental health researchers with few resources with a do-it-yourself process for developing and piloting their own Web-based app. The app development process outlined in this paper is intended to assist health researchers with limited resources to *pilot* an app to establish a proof of concept. This method may not be suitable, nor is recommended, for health researchers who (1) have funds to pay an app developer or (2) are conducting a large-scale trial that requires a more polished app than can be afforded using the proposed process. Readers who are interested in other mHealth platforms that can enable the creation of a mobile intervention should visit the Web pages for LifeGuide (University of Southampton, Southampton, UK) and Chorus (University of California, Los Angeles, California).

The steps presented are not exhaustive. Additional considerations should be stressed when applying the process presented in this paper. If researchers intend to collect personal health information from their participants, they must ensure that the survey platform they use is compatible for collecting and storing such information. Researchers are strongly encouraged to assess options at their own institutions and to use secure survey platforms (eg, REDCap) that are able to collect and store sensitive data. Both within the app itself and when posting their app on the Google Play store, researchers should include privacy policy and terms of use sections that explicitly state the conditions upon which users download and use the app, the expectations users should have when using the app, and a disclaimer regarding that the user acknowledges the conditions presented when using the app. This information increases transparency to users and offers protection for researchers. Finally, depending on the specific population and their needs, researchers may wish to adopt a co-design approach to designing and constructing their app interventions [39]. Specifically, to learn about the needs, attitudes, and preferences of their end users, health researchers may wish to conduct mixed method studies to understand how to best tailor an app intervention [40] to inform phase 1 (establishing a theoretical framework) or phase 2 (wireframe and content development) of the app building process [27-31]. Information regarding ways to better tailor the app to a specific population should account for the limitations of the survey platform being leveraged (eg, inability to facilitate live video conferencing).

The worldwide proliferation of personal-use mobile phones has provided mental and behavioral health researchers with an opportunity to deliver app-based interventions to those in need. Mobile phone apps are affordable, highly accessible, and capable of delivering empirically informed health interventions. For example, studies find that digital interventions that mirror the content of in-person therapy can perform just as well in reducing mood symptoms [41,42]. Mobile phone apps can be a tremendous resource for those in underserved areas, as the majority of mental and behavioral health specialists practice in densely populated regions, leading to a disparity in access to care for many ethnic minority individuals and those that reside in rural areas [43-45]. Although this paper used iCanThrive, a mental well-being intervention, to illustrate the process of building an app, the process outlined in this paper can in theory be used to create other types of health interventions. For example, health researchers who wish to build an app to reduce smoking may wish to use an app to deliver educational content on the harms of smoking. Researchers interested in diet and exercise may specify portions of an app to collect information on meals and activity levels.

### Limitations

The method described in this paper may not be applicable to developing app-based interventions for iOS (ie, Apple) devices. The iOS app store has strict guidelines for publishing apps and tend to only publish native apps. This process does not allow for fine-grained passive data collection that leverages mobile phone sensors. Finally, the app process described in this paper is not relevant for health researchers who are interested in developing an app that enables live video conferencing.

### Conclusions

Enabling researchers to cheaply and rapidly develop and test mobile interventions, through processes such as the one outlined in this paper, can ultimately lead to increased access to evidence-based behavioral and mental health interventions.

## Abbreviations

CBT: cognitive behavioral therapy
HIPAA: Health Insurance Portability and Accountability Act
mHealth: mobile health
REDCap: Research Electronic Data Capture
